# Peripheral Insulin Resistance and Alzheimer's Disease: Possible Mediators Including Extracellular Vesicles

**DOI:** 10.1111/ggi.70483

**Published:** 2026-04-13

**Authors:** Naotaka Izuo, Hiroshi Kondoh, Takahiko Shimizu

**Affiliations:** ^1^ Research Center for Advanced Science and Technology The University of Tokyo Tokyo Japan; ^2^ Geriatric Unit, Graduate School of Medicine Kyoto University Kyoto Japan; ^3^ Department of Food and Reproductive Function Advanced Research Juntendo University Graduate School of Medicine Tokyo Japan

**Keywords:** Alzheimer's disease, amyloid β, extracellular vesicles, insulin resistance, type 2 diabetes mellitus

## Abstract

Epidemiological studies consistently indicate that type 2 diabetes mellitus increases the risk of Alzheimer's disease (AD). Accumulating evidence suggests that insulin resistance, rather than hyperglycemia per se, is the principal metabolic factor associated with AD development. Population‐based longitudinal studies show that insulin resistance is strongly linked to earlier amyloid β (Aβ) accumulation, whereas its association with tau pathology remains inconsistent. Importantly, insulin resistance‐related cognitive decline and earlier disease onset cannot be fully explained by the extent of Aβ deposition alone, implying the involvement of additional pathogenic pathways. Animal studies further support a role for insulin resistance independent of sustained hyperglycemia. Genetic models of systemic insulin resistance demonstrate impairments in cognition, cerebral blood flow regulation, and emotional behavior, distinct from phenotypes observed in models with brain‐specific disruption of insulin signaling. Together with clinical observations showing preserved central insulin responsiveness in individuals with type 2 diabetes, these findings highlight peripheral insulin resistance as a key contributor to brain vulnerability in AD. How peripheral insulin resistance influences the brain remains incompletely understood. Emerging evidence suggests that extracellular vesicles may act as a possible mediator of peripheral–central communication by conveying bioactive molecules across tissues. In this review, we summarize epidemiological and experimental evidence linking peripheral insulin resistance to AD and discuss the potential, yet still speculative, role of extracellular vesicles in this process.

## Graphical abstract

This review discusses how type 2 diabetes mellitus may exacerbate Alzheimer's disease pathology, focusing on insulin resistance in peripheral organs and the potential mediating factors involved.

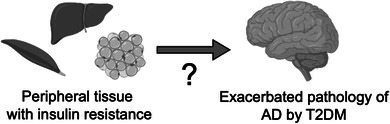

## 1 Introduction

Alzheimer's disease (AD) is a neurodegenerative disorder characterized by the accumulation of senile plaques composed of amyloid β (Aβ) and neurofibrillary tangles (NFTs) formed by hyperphosphorylated tau. At the time of clinical diagnosis, these pathological lesions and neuronal loss have already progressed substantially. Therefore, preventing disease onset is considered the most critical strategy for overcoming AD. Given that these pathological changes begin 10–20 years before clinical diagnosis [1, 2, 3], prevention can be conceptualized as the control of modifiable risk factors of AD and attenuation of its pathological processes. Furthermore, in the progressive stages of AD, Aβ deposited in cerebral vessels disrupts the structural and functional integrity of the blood–brain barrier (BBB) [4, 5]. Such vascular pathology contributes to increased BBB permeability and is strongly associated with the adverse events observed in anti‐amyloid therapies [6, 7]. In addition, both Aβ and tau actively induce and amplify neuroinflammatory responses, thereby accelerating the progression of AD pathology [8]. These interconnected processes highlight the multifaceted AD pathophysiology.

Type 2 diabetes mellitus (T2DM) has been consistently shown to increase the risk of AD in population‐based prospective studies and meta‐analyses. Early systematic reviews reported that diabetes is associated not only with all‐cause dementia but also specifically with AD [9]. Subsequent meta‐analyses focusing on prospective cohort studies quantitatively estimated the relative risk of AD in individuals with T2DM to be approximately 1.5–2.0 [10]. More recent updated reviews have reaffirmed the robustness of the association between T2DM and AD incidence [11].

Importantly, accumulating evidence indicates that among the pathophysiological components of T2DM, insulin resistance, rather than hyperglycemia per se, represents the principal risk factor for AD development. In the Hisayama Study, prospective follow‐up demonstrated that hyperglycemia during the 75‐g oral glucose tolerance test was associated with an increased future risk of AD, whereas fasting plasma glucose levels showed no significant association [12]. Similarly, the Rotterdam Study reported that elevated fasting insulin levels were linked to a higher incidence of AD within several years, whereas fasting glucose levels showed no such relationship [13]. Collectively, these findings support a consistent model in which insulin resistance accelerates or modifies key pathological processes underlying AD, thereby promoting earlier cognitive decline.

In this review, we aim to discuss the mechanisms by which insulin resistance exacerbates AD pathology. First, we summarize epidemiological evidence regarding how individual components of AD pathology are modified by T2DM. Next, we focus on studies using animal models to highlight mechanistic pathways that appear to be potentially critical for this AD exacerbation. Finally, we discuss possible mediators through which insulin resistance modifies brain pathology, including the involvement of extracellular vesicles (EVs).

## 2 Epidemiological Evidence for Insulin Resistance as a Risk Factor for AD

Epidemiological studies identifying insulin resistance as a risk factor for AD [12, 13], as discussed in Section 1, have generally diagnosed AD according to standardized clinical criteria such as the NINCDS–ADRDA guidelines, which define AD by the presence of memory‐centered, multidomain cognitive impairment accompanied by functional decline in activities of daily living. Focusing specifically on cognitive impairment, long‐term longitudinal studies—including the Framingham Offspring cohort and the Atherosclerosis Risk in Communities cohort—have consistently reported that elevated insulin resistance in midlife is associated with subsequent memory decline and brain atrophy later in life [14, 15].

**FIGURE 1:**
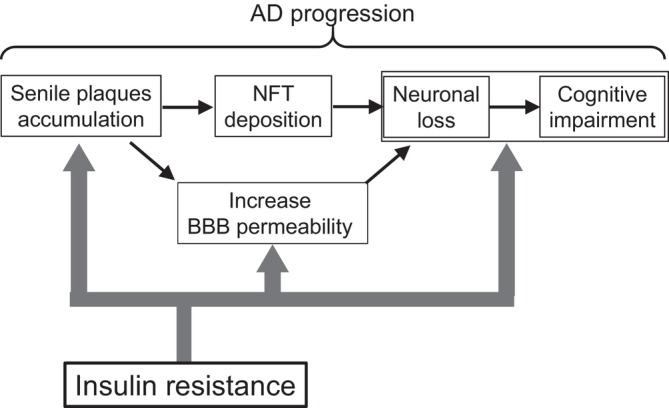
Target pathology of insulin resistance in AD pathology. In the epidemiological findings, insulin resistance is suggested to modify senile plaque accumulation and cognitive impairment accompanied by neuronal loss mediated by possibly different mechanisms represented by solid arrows. In contrast, evidence of the association of insulin resistance and tau pathology is inconsistent, represented by a dashed arrow. AD, Alzheimer's disease; NFT, neurofibrillary tangle.

A widely accepted framework for the core pathophysiology of AD posits a sequential process in which amyloid plaque accumulation acts as an initiating trigger, followed by the progression of tau pathology and, together with downstream processes such as BBB dysfunction and neuroinflammation, leads to subsequent neurodegeneration and cognitive decline. To better understand which pathological mechanisms of AD are influenced by insulin resistance, we first focus on its relationship with amyloid plaque accumulation. In the Hisayama Study, higher midlife HOMA‐IR was independently associated with increased amyloid plaque burden observed at brain autopsy approximately 15 years later, whereas fasting plasma glucose showed no such association [16]. Similarly, the Wisconsin Registry for Alzheimer's Prevention, which primarily enrolls cognitively normal middle‐aged adults with a family history of AD, demonstrated that among normoglycemic participants, elevated HOMA‐IR was significantly associated with Aβ PET positivity [17]. Moreover, similar findings have been reported in a Finnish population‐based cohort, in which midlife insulin resistance predicted increased Aβ PET signal decades later [18], further supporting a model in which insulin resistance acts at an early stage of AD pathogenesis by promoting initial Aβ accumulation (Figure 1).

In contrast, large‐scale prospective studies directly linking insulin resistance to tau pathology in the context of AD remain limited, and the evidence is less consistent. In the Hisayama Study, the association between midlife HOMA‐IR and NFT burden assessed by postmortem neuropathological analysis did not reach statistical significance [16]. Among cognitively normal adults, an association between elevated HOMA‐IR and increased cerebrospinal fluid (CSF) levels of total tau and phosphorylated tau has been reported [19], whereas this relationship was not observed in a more recent study [20]. In the Wisconsin cohort, HOMA‐IR was not significantly associated with entorhinal cortex tau PET signals, nor was there evidence of interaction with cerebral Aβ burden assessed by PiB PET. Furthermore, diabetes status was not associated with tau PET signal overall, suggesting that in middle‐aged to older adults at preclinical or very early stages of AD, insulin resistance may not be strongly linked to tau pathology [21] (Figure 1).

Interestingly, insulin resistance is associated with increased BBB permeability in patients with AD. In a cohort of nondiabetic AD patients, higher values of the triglyceride–glucose index, a marker of insulin resistance, were associated with increased CSF‐to‐serum albumin ratios and λ free light chain levels, both reflecting greater BBB permeability, without corresponding associations with Aβ or tau biomarkers [22]. These indicate that the relationship between insulin resistance and BBB permeability involves a pathway distinct from Aβ or tau (Figure 1).

Taken together, these findings indicate that insulin resistance shows a relatively consistent association with amyloid plaque accumulation, whereas its relationship with tau pathology remains less clear. This dissociation suggests that, in addition to its effects on amyloid pathology, insulin resistance may influence AD progression through additional mechanisms. In particular, emerging evidence linking insulin resistance to BBB dysfunction independently of Aβ or tau biomarkers raises the possibility that metabolic disturbances may also contribute to AD through pathways distinct from the canonical amyloid–tau cascade that drive neurodegeneration and cognitive decline (Figure 1).

## 3 Evidence From Animal Models of Insulin Resistance in the Context of AD

Epidemiological evidence has established insulin resistance as a major factor promoting the development of AD. To explore the mechanisms by which insulin resistance promotes AD pathogenesis, animal model‐based experimental approaches have been employed. Insulin resistance has most commonly been induced by high‐fat diet (HFD) feeding [23, 24]; however, this approach inevitably confounds the effects of insulin resistance with those of severe hyperlipidemia. Alternatively, pancreatic β cell destruction induced by streptozotocin administration [25] reduces insulin signaling, but the resulting persistent and extreme hyperglycemia makes it difficult to isolate the effects of insulin resistance per se on brain function. To overcome these limitations, genetically engineered models of insulin resistance have been developed.

Baba and colleagues generated knock‐in mice carrying a point mutation (P1195L) within the tyrosine kinase domain of the insulin receptor gene (IR‐KI mice) [26]. Homozygous mice die shortly after birth due to metabolic ketoacidosis, whereas heterozygous mice exhibit systemic insulin resistance throughout life without developing sustained hyperglycemia [26, 27]. By crossing these mice with an AD model that closely recapitulates prodromal AD pathology (APP knock‐in mice) [28, 29], a double knock‐in model (IR/APP‐dKI) was generated. This model allows evaluation of the impact of insulin resistance on AD‐related phenotypes while largely excluding the confounding influence of chronic hyperglycemia [30].

IR/APP‐dKI mice exhibited impairments in memory function and reduced stimulus‐induced cerebral blood flow responses in cortical regions, accompanied by decreased expression of nicotinic α7 acetylcholine receptors without affecting plaque deposition. Notably, these mice also displayed alterations in emotional behavior that are relevant to the neuropsychiatric and affective disturbances observed in AD [31]. These findings suggest that insulin resistance can induce memory impairment and emotional changes independently of sustained hyperglycemia. Importantly, the absence of amyloid plaque accumulation in this mouse model suggests that it allows isolation of insulin resistance‐related signals associated with cognitive impairment and emotional changes.

The phenotypes observed in IR/APP‐dKI mice differ in several respects from those reported in previous AD models with genetically induced insulin resistance. For example, IR/APP‐dKI mice exhibit increased depression‐like behavior together with reduced anxiety, whereas neuron‐specific insulin receptor knockout mice show increases in both depression‐ and anxiety‐like behaviors [32]. In another model, mice generated by crossing neuron‐specific insulin‐like growth factor 1 receptor (IGF‐1R) knockout mice—targeting a signaling pathway distinct from insulin receptor signaling—with AD model mice exhibited improvements in both Aβ pathology and cognitive function [33]. Given that IR/APP‐dKI mice exhibit insulin resistance in peripheral organs as well as in the brain, these observations suggest that peripheral insulin resistance and central nervous system insulin resistance may exert qualitatively distinct effects on the brain. In particular, signals arising from peripheral insulin resistance may contribute to the exacerbation of AD pathology in patients with T2DM.

## 4 Humoral Factors Potentially Mediating Modifications of AD by Peripheral Insulin Resistance

**FIGURE 2:**
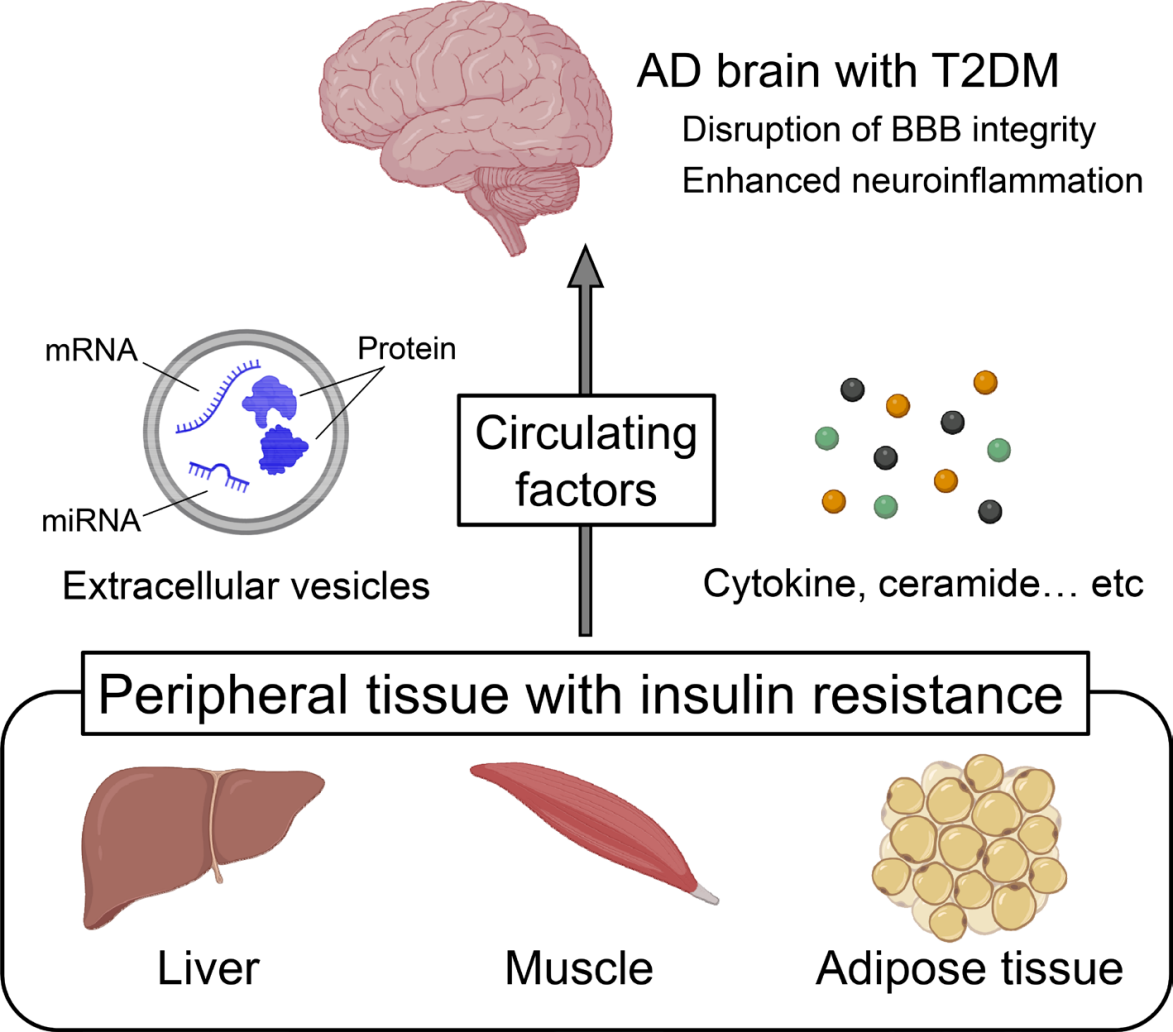
Under AD patients with T2DM, circulating factors, such as cytokines, ceramide, and extracellular vesicles including nucleic acids and proteins, are secreted from peripheral tissues with insulin resistance and affect the brain. They modify AD pathology by disrupting BBB integrity and enhance neuroinflammation. AD, Alzheimer's disease; BBB, blood–brain barrier; T2DM, type 2 diabetes mellitus. Image was drawn with support of BioRender.

In T2DM, insulin resistance in peripheral organs such as adipose tissue, liver, and skeletal muscle is accompanied by chronic inflammation, with increased circulating levels of humoral factors including tumor necrosis factor‐α (TNF‐α) and interleukin‐6 (IL‐6). These inflammatory cytokines are produced primarily by adipose tissue macrophages, adipocytes, and hepatic macrophages, and negatively regulate downstream signaling of the insulin receptor, thereby inducing and exacerbating insulin resistance [34, 35]. In organs with advanced insulin resistance, the expression of TNF‐α and IL‐6 is further upregulated, leading to the establishment of a vicious cycle of chronic low‐grade inflammation [34, 35] (Figure 2).

Circulating TNF‐α and IL‐6 do not cross the BBB in large amounts to act directly on neurons. Instead, these cytokines interact with brain microvascular endothelial cells, leading to increased BBB permeability and impaired barrier integrity. These effects are mediated through mechanisms including reduced expression or redistribution of tight junction proteins (such as claudin‐5, occludin, and ZO‐1), increased production of reactive oxygen species, and activation of matrix metalloproteinases (MMPs) [36, 37]. Disruption of BBB integrity and increased permeability allow plasma proteins (including albumin and fibrinogen) and peripheral cytokines to leak into the brain parenchyma, where secondary inflammatory responses are propagated through activation of microglia and astrocytes. In the clinical evidence, higher levels of insulin resistance were associated with greater BBB permeability, assessed by the serum‐to‐CSF albumin ratio, in patients with AD [22]. Elevated peripheral inflammatory cytokines associated with insulin resistance and the resulting impairment of BBB integrity are therefore likely to contribute, at least in part, to the exacerbation of AD by promoting the propagation of inflammation into the brain (Figure 2).

Ceramides are non‐inflammatory humoral mediators derived primarily from the liver and adipose tissue. Some clinical and cohort studies have demonstrated that circulating and tissue ceramide levels are significantly elevated in T2DM and insulin‐resistant states [38, 39]. Owing to their lipophilicity, ceramides can cross the BBB and reach the brain, where they have been suggested to impair neuronal energy metabolism and promote apoptosis [40]. Furthermore, prospective cohort studies in older women have reported that higher baseline serum ceramide levels are associated with a significantly increased risk of subsequent AD onset [41], suggesting that ceramides may act as mediators through which peripheral insulin resistance exacerbates AD pathology (Figure 2).

EVs mediate intercellular and inter‐organ communication by transferring cargo from donor cells to recipient cells, thereby transmitting biological signals. Freeman and colleagues [42] investigated changes in the particle number, cargo, and function of circulating EVs in T2DM by integrating cross‐sectional and longitudinal human cohorts with in vitro experiments. They demonstrated that circulating EV concentrations were significantly higher in individuals with T2DM than in healthy controls and positively correlated with HOMA‐IR, suggesting that insulin resistance itself enhances EV secretion (Figure 2).

Yang and colleagues identified 13 microRNAs that were characteristically increased in adipose tissue–derived EVs from patients with T2DM and demonstrated, using Mendelian randomization analysis, that increased miR‐125a‐5p causally elevates AD risk [43]. In large‐scale validation analyses, higher levels of miR‐125a‐5p in serum EVs were associated with an increased risk of amnestic mild cognitive impairment (aMCI), reduced left hippocampal volume, and showed high discriminative performance for aMCI. Other small cross‐sectional studies have likewise reported increased levels of serum miR‐125a‐5p in patients with AD [44]. At the same time, miR‐125a‐5p has been reported to exert protective functions, including anti‐inflammatory effects [45]. Whether these protective actions are directly relevant to AD pathology, or instead reflect other biological effects of miR‐125a‐5p, remains to be determined. Given that miR‐125b‐5p, which shares a similar seed sequence with miR‐125a‐5p, has been shown to suppress synaptic transmission by destabilizing dendritic spine structures [46], miR‐125a‐5p may also exert inhibitory effects on neuronal function and act in a disease‐promoting manner in the chronic context of AD. In addition to the miRNAs showing increased expression, Yang et al. also identified 44 miRNAs that were decreased in T2DM [43], among which molecules involved in the maintenance of brain function may be included, raising the possibility that their reduction contributes to AD progression.

In addition, Ying and colleagues reported that miR‐155 contained in EVs derived from adipose tissue–resident macrophages was associated with insulin resistance [47]. miR‐155 promotes proinflammatory cascades while suppressing anti‐inflammatory pathways [48], and its elevation has been observed even before the establishment of insulin resistance in both patients and disease models. Furthermore, miR‐155 has been predicted by machine‐learning approaches as part of the shared molecular background of T2DM and AD [49], and its increased expression in the brains of AD patients is well documented [50].

Collectively, these findings suggest that in T2DM, humoral factors and EVs derived from multiple peripheral organs—including adipose tissue—may contribute to the amplification of AD pathology through BBB dysfunction and neuroinflammation. Although the extent to which peripheral organ–derived EVs can cross the BBB and reach the brain parenchyma remains incompletely understood, reduced BBB integrity in AD suggests that their brain entry may be enhanced compared with healthy conditions. Moreover, disease‐dependent alterations in EV properties may enable the acquisition of brain‐targeting capabilities. Comprehensive analyses will therefore be required to clarify the role of EVs in the exacerbation of AD pathology driven by peripheral insulin resistance (Figure 2).

## 5 Conclusion and Perspective

In this review, we comprehensively summarized epidemiological, experimental, and molecular evidence addressing how peripheral insulin resistance in T2DM may contribute to the onset and progression of AD. Epidemiological studies consistently demonstrate an association between insulin resistance and Aβ accumulation, whereas the relationship between insulin resistance and tau pathology remains unclear. Importantly, insulin resistance–associated cognitive decline in AD likely reflects not only its effects on Aβ pathology, but also additional pathological processes through which insulin resistance modulates disease progression. In parallel, consideration of findings across different animal models suggests the importance of peripheral insulin resistance in contributing to the exacerbation of AD‐related phenotypes. Against this background, several peripheral factors have emerged as potential candidates mediating the effects of peripheral insulin resistance on the brain, including inflammatory cytokines, ceramides, and EVs. Chronic low‐grade inflammation associated with peripheral insulin resistance has been linked to impairment of BBB integrity, which may facilitate the propagation of inflammatory signals from the periphery into the brain. In this context, an increasing number of findings indicate that EVs are altered in T2DM, with changes in their abundance, molecular cargo like miRNAs, and biological effects that correlate with insulin resistance. Such alterations raise the possibility that EVs may act as mediators linking peripheral insulin resistance to central nervous system vulnerability.

Future studies should aim to integrate peripheral insulin resistance‐driven mechanisms across multiple levels, including Aβ pathology, BBB function, neuroinflammation, and EV‐mediated inter‐organ signaling, to better understand how these processes collectively contribute to AD pathogenesis.

From a therapeutic perspective, peripheral insulin resistance represents a potentially modifiable factor during the preclinical and prodromal stages of AD, and metabolic interventions may indirectly influence brain vulnerability. In this context, EVs warrant further investigation not only as mediators of disease processes and indicators of pathological states or therapeutic responses, but also as potential therapeutic targets or modulators linking peripheral metabolic dysfunction to central neurodegeneration. A framework that connects peripheral metabolic alterations with brain pathology may provide important insights for refining preventive and interventional strategies in AD.

## Ethics Statement

The authors have nothing to report.

## Conflicts of Interest

The authors declare no conflicts of interest.

## Data Availability

The data that support the findings of this study are available on request from the corresponding author. The data are not publicly available due to privacy or ethical restrictions.
